# Field Measurements and Numerical Simulations of Temperature and Moisture in Highway Engineering Using a Frequency Domain Reflectometry Sensor

**DOI:** 10.3390/s16060857

**Published:** 2016-06-10

**Authors:** Yong-Sheng Yao, Jian-Long Zheng, Zeng-Shun Chen, Jun-Hui Zhang, Yong Li

**Affiliations:** 1 School of Traffic & Transportation, Changsha University of Science &Technology, Changsha 410114, China; yyaoai@connect.ust.hk (Y.-S.Y.); zjl@csust.edu.cn (J.-L.Z.); zjhseu@csust.edu.cn (J.-H.Z.); 2 Department of Civil and Environmental Engineering, The Hong Kong University of Science and Technology, Clear Water Bay, Kowloon, Hong Kong, China; 3 Shenzhen Bridge-Doctor Design & Research Institute Co., Ltd., Shenzhen 518048, China; liy2000@163.com

**Keywords:** FDR sensor, highway reconstruction and extension, temperature and moisture, field measurement, numerical simulation

## Abstract

This paper presents a systematic pioneering study on the use of agricultural-purpose frequency domain reflectometry (FDR) sensors to monitor temperature and moisture of a subgrade in highway extension and reconstruction engineering. The principle of agricultural-purpose FDR sensors and the process for embedding this kind of sensors for subgrade engineering purposes are introduced. Based on field measured weather data, a numerical analysis model for temperature and moisture content in the subgrade’s soil is built. Comparisons of the temperature and moisture data obtained from numerical simulation and FDR-based measurements are conducted. The results show that: (1) the embedding method and process, data acquisition, and remote transmission presented are reasonable; (2) the temperature and moisture changes are coordinated with the atmospheric environment and they are also in close agreement with numerical calculations; (3) the change laws of both are consistent at positions where the subgrade is compacted uniformly. These results suggest that the data measured by the agricultural-purpose FDR sensors are reliable. The findings of this paper enable a new and effective real-time monitoring method for a subgrade’s temperature and moisture changes, and thus broaden the application of agricultural-purpose FDR sensors.

## 1. Introduction

Moisture content is an important physical parameter when we study soil properties, which will change with the variation of moisture content. Monitoring the variation of moisture content in the field of civil engineering projects, such as highway subgrades, railway embankments, prevention and treatment of slopes, foundation engineering, is an important research direction.

The soil-filled structure for the highway subgrade is compacted in layers according to specific technical standards, and the moisture content and temperature changes in the soil have important influences on subgrade performance (modulus and settlement) [1]. As subgrade soil is unsaturated soil, there exists a certain correlation among its moisture, temperature and modulus: the moisture and temperature have a substantial effect on the modulus, especially the change of moisture content significantly affect the modulus, sometimes even plays a decisive role [2,3,4,5,6]; moreover, the moisture and temperature are also main factors influencing the settlement deformation of the subgrade [7,8]. The variation of moisture and modulus can impact the pavement structure and further affect the traffic safety on and functions of the highway [9]. Previous researchers have done extensive laboratory tests on modulus and settlement changes with the variation of moisture and temperature, but there is no appropriate method for real-time monitoring of moisture content and temperature changes of subgrade soil in the field. In most of the previous studies on highway construction or reconstruction/extension projects, the moisture content and temperature in the subgrade are subject to numerical simulation and calculation based on climatic parameters (temperature, moisture, wind speed, rainfall, evaporation and transpiration, underground water level, *etc.*) [10,11,12,13]. Though certain effectivity has been achieved, the method requires many parameters, parameter acquisition is difficult and the calculations are complex [14]. Therefore, an appropriate and effective method for real-time monitoring of soil moisture and temperature is imperative.

At present, moisture content can be detected by both direct and indirect methods. Direct methods are mainly oven-drying methods, while indirect methods include the resistor method, ray method, tensiometer method and recently developed methods such as frequency domain reflectometry (FDR).

FDR is used to test the apparent dielectric constant of soil based on the principle of electromagnetic pulses and according to electromagnetic wave propagation in the soil, and then to obtain the volumetric moisture content in the soil. Hilhorst presented a frequency domain decomposition method [15] after carrying out a large amount of research and proposed that the dielectric constant of soil can be decomposed at a reasonable test frequency. This method is to obtain the soil’s conductivity from the imaginary part of the dielectric constant at a reasonable frequency (20–30 MHz) by means of the vector voltage measurement technique, and then to convert it into the soil’s moisture content from the real part of the dielectric constant. Moreover, he designed and developed a special Application Specific Integrated Circuit (ASIC) chip for soil moisture sensors used in FDR, which not only improved the reliability of FDR soil moisture sensors but also greatly reduced the production cost, thus enabling the production and promotion of FDR soil moisture sensors [16]. Leib *et al.* [17] found that uncalibrated FDR sensors can be used for observing the trend of soil moisture content, but cannot obtain accurate absolute moisture content, so their calibration parameters should be determined accurately in advance for different soil types. Tejero *et al.* [18] studied the measurement of soil moisture content using FDR sensors in two ways: direct drilling and conventional tillage, and found direct drilling to be more accurate.

The oven-drying method is of the most accurate when measuring soil moisture content, but in the field of civil engineering, particularly in highway engineering, this method will destroy the subgrade structure, so it is not allowed during the operation of the highway. Soil moisture sensors based on FDR technology are advantageous for their features such as cheapness, speed, easy handling and less soil disturbance, and are one of the common instruments for measuring *in situ* soil volumetric moisture content [19]. Ye *et al.* [20] and Choi *et al.* [21] studied the application of FDR sensors in environmental testing and evaluation, reaching the conclusion that they were effective in the field of environmental engineering. Skierucha *et al.* [22] studied the use of FDR for measuring the water content in different frequency ranges. Wilczek *et al.* [23] examined the monitoring of soil pore water salinity using an FDR sensor working at various frequencies up to 500 MHz. Gastao *et al.* [24] measured the irrigation schedules of cabbages by using a sub-hourly frequency-domain reflectometer. Girón *et al.* [25] studied the differences in physiological response between leaves and fruits of table olive trees under moderate water stress using FDR sensors. In summary, FDR technology can be considered a mature testing technique, widely applicable in agriculture and environmental engineering [26,27,28,29].

To sum up, as a technology for rapidly detecting the soil temperature and moisture content, FDR sensors have widely been used in agriculture, but studies on their application in civil engineering, especially in highway subgrade construction, reconstruction and extension are rarely found. In this paper, based on the testing principle of FDR sensors, extensive laboratory calibrations for soils were carried out first using FDR and then we present an application of FDR sensors in subgrade widening (including the old and newly-filled subgrades) and a new installation method for FDR sensor probes is proposed. A small weather station and GPRS wireless data transmission system were established in the field, and the real-time temperature and moisture content data monitored by FDR sensors are compared with the subgrade soil data obtained through numerical calculation to demonstrate the good real-time monitoring effect of FDR sensors, thus widening their application scope and providing reference values for other similar projects in the civil engineering field.

## 2. Field Measurement Based on Agricultural-Purpose FDR Sensor

### 2.1. Basic Composition and Testing Principle of FDR Sensor

#### 2.1.1. Basic Composition

**Figure 1:**
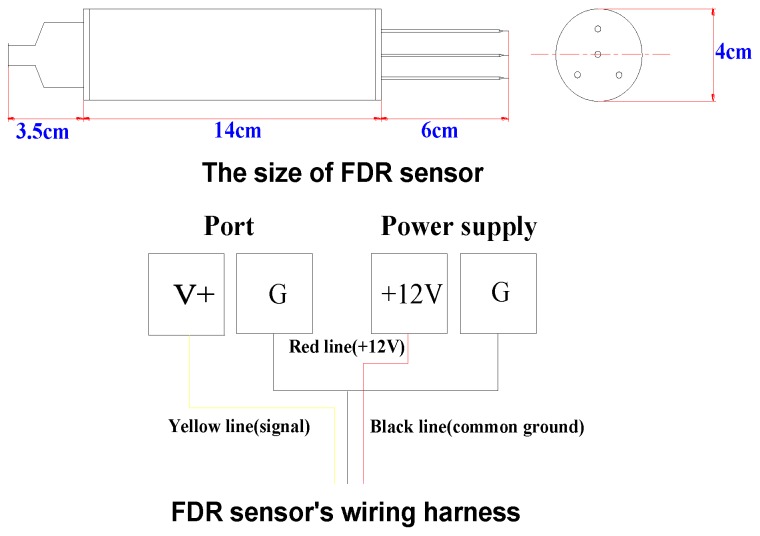
Dimensions of the FDR sensor.

The circuit of an FDR sensor is encased in a cylindrical cavity with a waterproof seal: four stainless steel needles on one end of the cavity form a sensing element, with the central needle serving as the main sensing terminal, and the three surrounding needles connected with one metal ring inside the cavity to form a common ground terminal. The physical dimensions of an FDR sensor are shown in Figure 1. When installing the sensor, its needles should be inserted into and in close contact with the medium to be tested, and its sensing range is within the cylinder bound by the three surrounding needles.

#### 2.1.2. Testing Principle

There is a specific functional relationship between the dielectric constant of the tested medium and the moisture content and temperature in the medium. Any changes in the dielectric constant are output as changes in DC voltage in the circuit through a data link, and the output data are received by a receiving terminal. The mechanism of measuring moisture content and temperature by the FDR sensor involves obtaining the dielectric constant of the soil. The moisture content and temperature can then be deduced from their calibrated relationship with the dielectric constant.

After taking the soil samples from field, the authors calibrated the FDR sensors in the soil samples in laboratory and obtained the relationships between voltage and volumetric water content, temperature. The calibration results are shown in Table 1. The technical parameters of FDR sensor are given in Table A1 at Appendix A.

### 2.2. Embedding of an Agricultural-Purpose FDR Sensor

#### 2.2.1. Project Overview

The case study is based on the Nanchang-Zhangshu Expressway Reconstruction and Extension Project in China. The old expressway, a bi-directional, four-lane road with an asphalt concrete pavement and a 27 m-wide subgrade, was put into service in December 1997. The widening project commenced in November 2012. After over a decade of operation, the the Nanchang-Zhangshu Expressway’s old subgrade is almost stably settled and it was found through field testing that the old subgrade soil moisture was higher than the filling initial moisture content, and the modulus of soils displayed a corresponding reduction. When the subgrade is widened, differential settlement may occur at the junctions between the new and old subgrades due to their differences in temperature, moisture content and compaction uniformity. Therefore, how to monitor and control the differential settlement is a key problem for the Nanchang-Zhangshu Expressway Reconstruction and Extension Project.

#### 2.2.2. Embedding Method and Process

**Figure 2:**
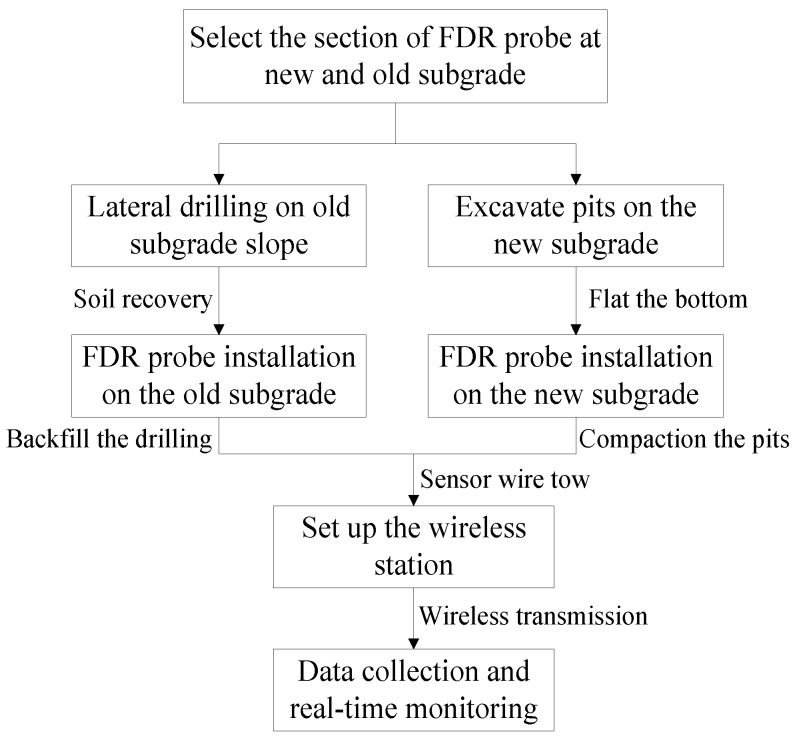
Flow diagram of FDR sensor installation.

**Figure 3:**
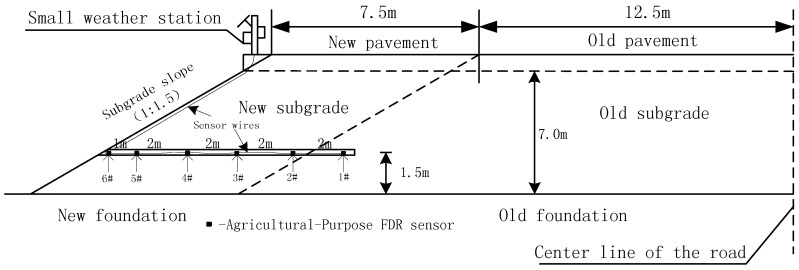
Embedding sketch for an agricultural-purpose FDR sensor.

There is no previous experience in embedding FDR sensors into a subgrade, and after the FDR sensors are embedded, the excavation will be filled with water and compacted (by road rollers), so in case of improper embedding or protection, FDR sensors will be damaged or large measurement errors will result. Given the above factors, the following methods are proposed in this paper:

(1) Embedding FDR sensor in old subgrade: drill horizontally to the predetermined depth at the side slope of the old subgrade with a drilling machine (borehole diameter: 11.0 cm); install the FDR sensor on one end of the PVC tube (diameter: about 5 cm), and push it to the bottom of the borehole from the other end of the PVC tube to make it completely and closely inserted into soil horizontally (in the old subgrade).
(2) Embedding FDR probes in new subgrade: at the preset subgrade elevation, compact and level the subgrade surface and excavate a pit (L × W × D: 40 cm × 30 cm × 20 cm) manually at a certain interval; then closely insert the probe into soil along the bottom of the pit in a lateral direction (the inserting direction is vertical to the longitudinal direction of the subgrade); last, excavate a small groove along the cross section of the newly-filled subgrade (W: 10 cm; D: 20 cm; L: 750 cm (viz. the width of the entire newly-filled subgrade)) to embed the sensor wire into the groove and lead it to the outer side of the subgrade slope. The embedding flow diagram and field embedding sketch for FDR sensors are shown in Figure 2 and Figure 3, respectively.

### 2.3. Data Acquisition and Transmission

**Figure 4:**
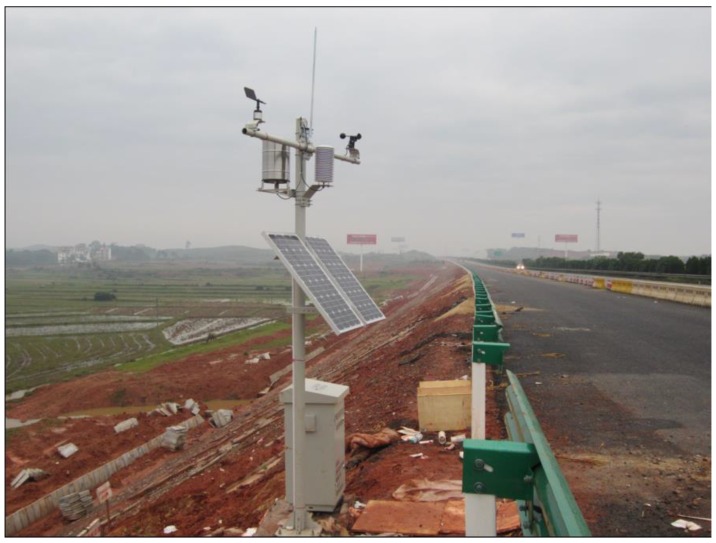
Small weather station (site picture).

After the new subgrade is filled, the wire of the FDR sensor should be led to the top of the subgrade slope with great care and be protected effectively to prevent damages (the wire is embedded at a depth about 20 cm below the surface of the subgrade slope). To monitor the changes in moisture content and temperature of the soil mass inside the subgrade and changes in temperature and moisture surrounding the subgrade in real-time, for this study, a small weather station is installed on the upper position of the side slope, with the support bar of the station placed in concrete in the substrate (Figure 4). On the support bar, an air moisture sensor, a rain sensor, a wind speed sensor, a wind direction sensor and a lightning rod are installed; on the side of the support bar, a meter box is installed, inside which is an FDR sensor data acquisition unit, a meteorological parameter data acquisition unit, a battery and a GPRS wireless transmission module, *etc.*, which enables real-time data to be monitored remotely. The acquisition unit acquires data every two hours and stores it in the GPRS module. Through the software installed in the computer that is compatible with the GPRS module, data stored in the GPRS module can be checked or received remotely at any time.

## 3. Numerical Simulation Based on Measured Data

Numerous factors influence the temperature and moisture of the soil mass inside the subgrade. The distributions of temperature and moisture of the subgrade soil are calculated assuming that the subgrade surface can be well drained and referencing the numerical calculation method for the balanced moisture proposed by Zhang and Zheng from the Changsha University of Science and Technology [30]. This numerical calculation method is a model with a comparatively comprehensive theoretical basis, because the influences exerted by the climate, soil quality, groundwater, and slope vegetation layer and upper structural layer are all thoroughly taken into account based on a thermo-hydro-mechanical coupling analysis of unsaturated soil. The parameters required for the numerical simulations are listed in Table 2.

### 3.1. Theoretical Model and Boundary Conditions

#### 3.1.1. Numerical Model

As the subgrade slope is subject to influences from rainfall, evaporation and transpiration, groundwater and other environmental factors, changes in its internal temperature and moisture are a typical hydro-thermo coupling process of a non-isothermal flow. Philip and De Vries, according to the coupled water-vapor-heat migration theory based on energy and mass, proposed the gas-liquid flow motion model influenced by water and heat gradients [31], which is expressed as follows:

(1) $$\frac{1}{\rho_{w}}\frac{\partial }{\partial x}\left(D_{v}\frac{\partial P_{v}}{\partial x}\right)+\frac{1}{\rho_{w}}\frac{\partial }{\partial y}\left(D_{v}\frac{\partial P_{v}}{\partial y}\right)+\frac{\partial }{\partial x}\left(k_{x}\frac{\partial (\psi +y)}{\partial x}\right)+\frac{\partial }{\partial y}\left(k_{y}\frac{\partial (\psi +y)}{\partial y}\right)+Q=\lambda \frac{\partial (\psi )}{\partial t}$$

(2) $$L_{v}\frac{\partial }{\partial x}\left(D_{v}\frac{\partial P_{v}}{\partial x}\right)+L_{v}\frac{\partial }{\partial y}\left(D_{v}\frac{\partial P_{v}}{\partial y}\right)+\frac{\partial }{\partial x}\left(\lambda_{tx}\frac{\partial T}{\partial x}\right)+\frac{\partial }{\partial y}\left(\lambda_{ty}\frac{\partial T}{\partial x}\right)+Q_{t}=\lambda_{v}\frac{\partial (\psi )}{\partial t}$$

where: *ρ_w_*—water density; *P_V_*—vapor pressure of water in soil; *D_V_*—diffusion coefficient of vapor in soil pores; *k_x_*, *k_y_*—infiltration coefficient of soil in horizontal and vertical directions; *ψ*—soil suction; Q—boundary water flow; *λ*—slope of the soil-water characteristic curve; y—position head; *L*_v_—latent heat of water evaporation; *λ_v_*—volumetric heat capacity of soil, *λ_tx_*, *λ_ty_*—thermal conductivity of soil mass in horizontal and vertical directions; *Q_t_*—boundary heat flux; T—absolute temperature.

Based on the variables *ψ* and *P_V_* in the above control equation and the equation proposed by Edlefsen and Anderson [32], the following relation can be established:

(3) $$P_{v}=P_{vs}(e^{-\frac{\psi w}{\rho_{w}RT}})=P_{VS}h_{air}$$

where: *P_VS_*—saturated vapor pressure; *w*—gram-molecular weight (GMW) of vapor; *h_air_*—relative humidity of air; *R*—gas content. Other symbols have the meaning as stated above.

#### 3.1.2. Calculation Equation for Flow Boundary Condition

The flow boundary condition is determined by rainfall and evaporation:

(4) $$Q=P_{r}-E$$

The flow boundary is water exchanges mainly in the form of rainfall and evaporation and controlled by importing meteorological parameters. In the equation, rainfall *P_r_* is the value measured by the small weather station. Since the water supply is limited, the continuous evaporation rate gradually decreases. To this end, Wilson has derived the Wilson-Penman evaporation model [33] to calculate actual evaporation on the soil surface in combination with the change in relative humidity of the soil:

(5) $$E=\frac{\Delta R_{n}+\eta E_{a}}{\Delta +\eta H}$$

where: *E*—actual evaporation intensity; *R_n_*—net radiation on soil surface; *η*—humidity constant; ∆—slope of saturated-vapor-pressure -and-temperature curve; *E_a_*—potential evaporation intensity, *E_a_* = *f*(*u*) *P_gw_* (*I* − *H*); *f*(*u*)—wind function, *f*(*u*) = 0.35 × (1 + 0.15*u*); *P_gw_*—vapor pressure of air on soil surface; *u*—wind speed of soil surface; *H* = 1/*R_h_*—reciprocal of relative humidity of soil surface; *I = 1/h_B_*—reciprocal of relative humidity of air.

#### 3.1.3. Temperature Boundary Conditions

The subgrade slope surface temperature condition equation [34] in the Wilson formula is used again:

(8) $$T_{s}=T_{a}+\frac{1}{\eta f\left(u\right)}\left(R_{n}-E\right)$$

where: *T_S_*—soil surface temperature (the climate state at the starting point of the simulation is set as the initial soil temperature); *T_a_*—soil surface air temperature; other symbols have the meaning as stated above.

Meteorological parameters such as rainfall, air temperature, wind speed and relative moisture are obtained from the small weather station on the upper position of the subgrade slope; the parameters for hydraulic and thermal properties of soil, including those for the soil-water characteristic curve model and the functions of the unsaturated infiltration coefficient, thermal conductivity coefficient and volumetric heat capacity, can all be derived through tests.

### 3.2. Geometric Model for Finite Element Calculation

**Figure 5:**
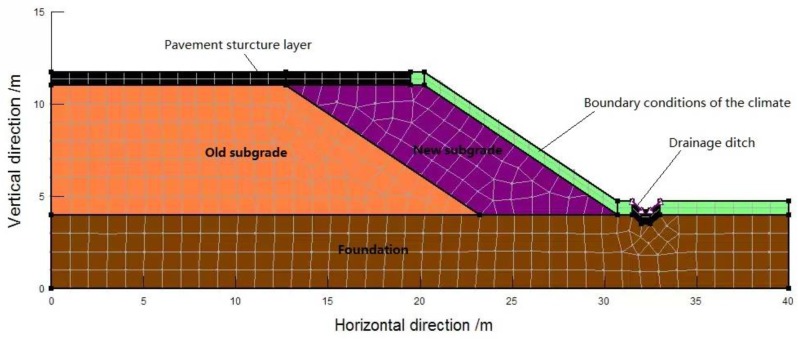
Numerical calculation model for the temperature and moisture of old and new subgrades.

The actual subgrade section size of the project case is taken as the calculation model, as shown in Figure 5: the embankment height, top surface width (semi amplitude) and groundwater depth are respectively 7.0, 20 and 1 m and the pavement structure layer is assumed to be impermeable and the central strip and drainage ditch to have good performance. A total of six monitoring points for FDR sensors are set up in total in the old and new subgrades (Figure 3). Climatic parameters are the values measured by the small weather station. Data acquisition started on 10 November 2013 and ended on 16 December 2015, spanning 767 days. In finite element simulation, every 5 days constitute a computational step, so 154 steps are demarcated in total. The hydraulic and thermodynamic parameters of the subgrade materials are acquired by means of the soil-water characteristic curve and infiltration test, as shown in Table 1. The numerical model is presented in Figure 5.

#### 3.2.1. Measured Climatic Parameters

**Figure B1:**
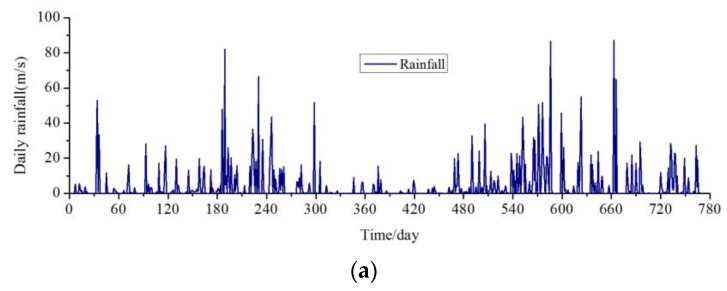

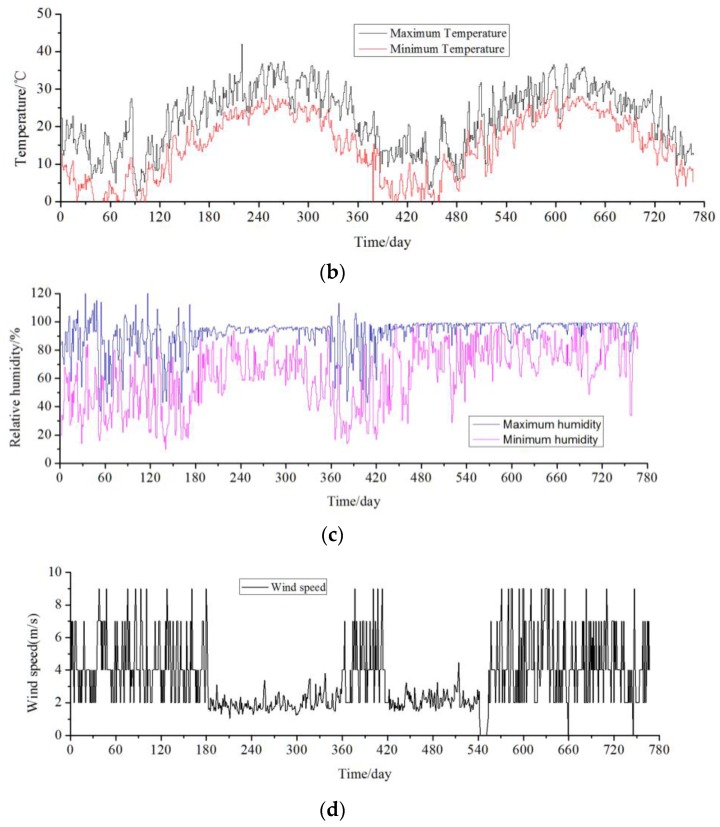
AReal-time Monitoring Climatic Parameters: (**a**) Daily Rainfall; (**b**) Daily Maximum and Minimum Air Temperatures; (**c**) Daily Humidity; (**d**) Daily Average Wind Speed.

The climatic parameters are acquired from the small weather station built in the field and transmitted remotely to the computer through the GPRS module for real-time acquisition or checks. In this paper, the climatic parameters in the project case were acquired from 10 November 2013 to 16 December 2015, spanning a total of 767 days. For convenience of plotting, it is assumed that 10 November 2013 is the first day of the test. During this period, meteorological parameters such as rainfall, atmospheric temperature, relative moisture and average wind speed were acquired every two hours. The daily average values of the acquired meteorological parameters change with time as shown in Figure B1 at Appendix B.

#### 3.2.2. Other Parameters

The soil-water characteristic curve of the subgrade soil (low liquid-limit clay) can be obtained using Fredlund and Xing’s model [35] through indoors experiments. Based on this curve, parameter fitting can be conducted to the relation between the suction of low liquid-limit clay and volumetric moisture content. The fitting results are given in Table 3. Fredlund and Xing’s model can be expressed as:

(9) $$\theta =C\left(s\right)\frac{\theta_{s}}{{\left\{\ln\left[e+{\left(s/a\right)}^{n}\right]\right\}}^{m}}\;,\;C\left(s\right)=1-\frac{\ln\left(1+s/s_{\gamma }\right)}{\ln\left(1+1000000/s_{\gamma }\right)}$$

where: *θ*—volumetric moisture content; *θ_s_*—saturated volumetric moisture content; *s*—suction; *s_y_*—matric suction corresponding to the residual moisture content; *a*—parameter reflecting the knee of the curve; *n*—parameter reflecting the slope of the straight segment on the curve; *m*—parameter reflecting the residual moisture content.

The infiltration coefficients of the subgrade and low liquid-limit clay are obtained through an indoor infiltration test, the thermal conductivity coefficient can also be obtained through an indoor test and calculation, the volumetric heat capacity and the physiological data of vegetation are values shown in [13]. The main parameters derived from the numerical calculation are given in Table 4.

### 3.3. Calibration for the Finite Elements

**Figure 6:**
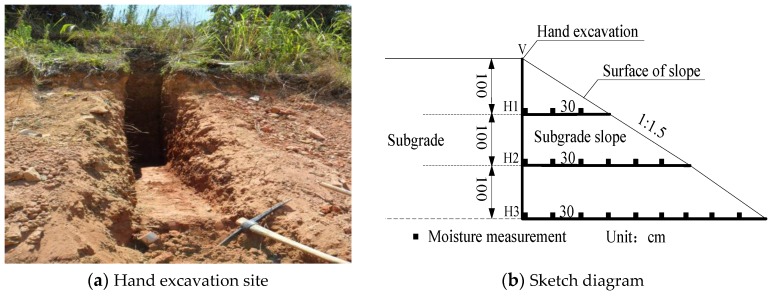
Hand excavation and sketch diagram. (**a**) Hand excavation site; (**b**) Sketch diagram.

In order to verify the numerical calculation, calibration is conducted through numerical calculation and field measurement values. The calibration is carried out by the following method: before the construction of the support structure, hand excavation is conducted on the subgrade slope of the existing road. The hand excavation will be carried out along the vertical excavation line (as shown in Figure 6a), V plane is the vertical plane and the planes H1, H2 and H3 are set at a distance of 1.0, 2.0 and 3.0 m, respectively, from the top of the subgrade (as shown in Figure 6b). A moisture content test (by the oven-drying method) will be conducted at the interval of 30 cm on plane H in the excavation to acquire the field measurement data of moisture content at each point.

**Figure 7:**
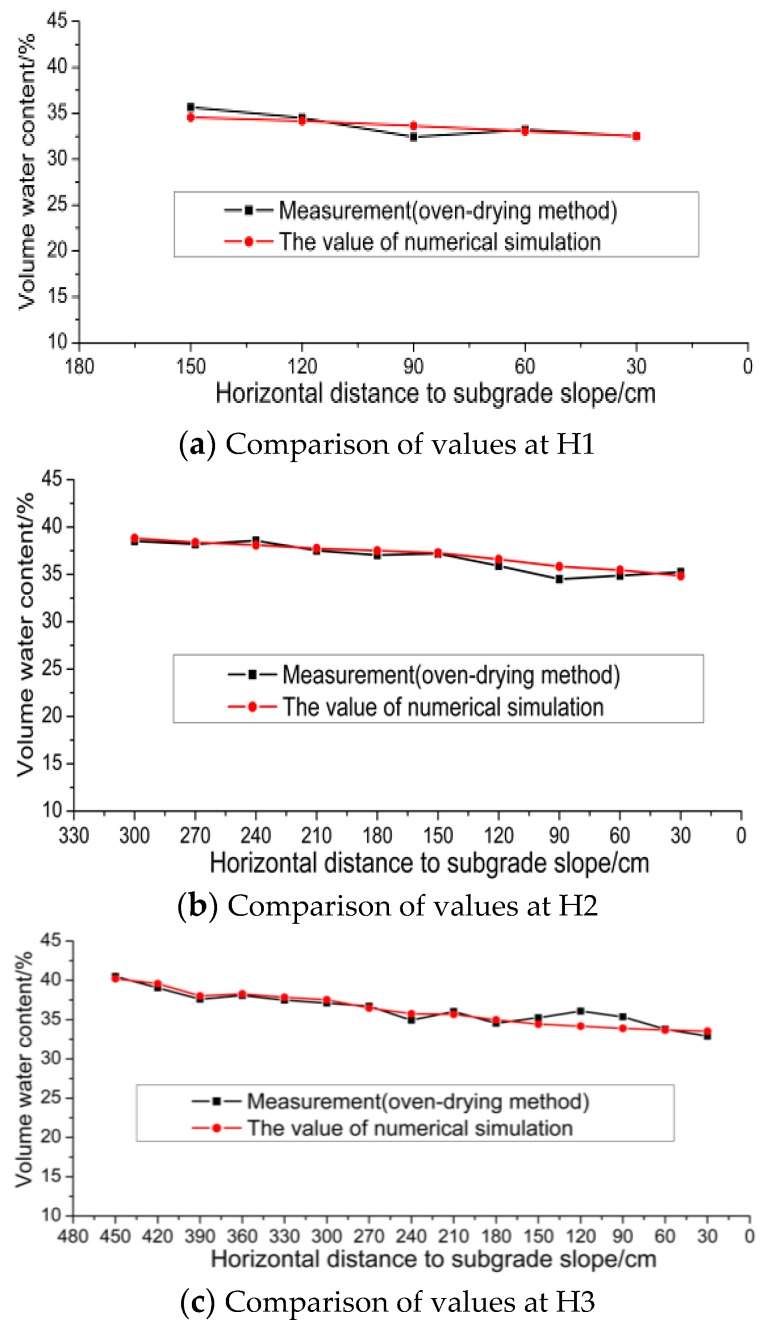
Comparison of measured and numerical calculated values. (**a**) Comparison of values at H1; (**b**) Comparison of values at H2; (**c**) Comparison of values at H3.

Numerical simulation is performed based on the theoretical basis and material parameters by using the local climate data in the period from the implementation of this section to the day of excavation. The results are shown in Figure 7.

Figure 7 shows that when the horizontal distance between the measuring point in the subgrade and the surface of the subgrade slope is larger than 150 cm, the computed value is close to that measured value and their average differences on planes H2 and H3 are 0.86% and 1.11%, and although due to the influence of the climate conditions, the measured values vary more significantly at the location with a level distance less than 1.5 m from subgrade slope, the average difference between the calculated and measured values on H1, H2 and H3 is 1.69%. Therefore, the results of numerical simulation agree well with those of the field measurements. The above calibration proves the validity of the numerical calculation method selected in the paper.

## 4. Measured Results and Analyses

### 4.1. Temperature Observations

**Figure 8:**
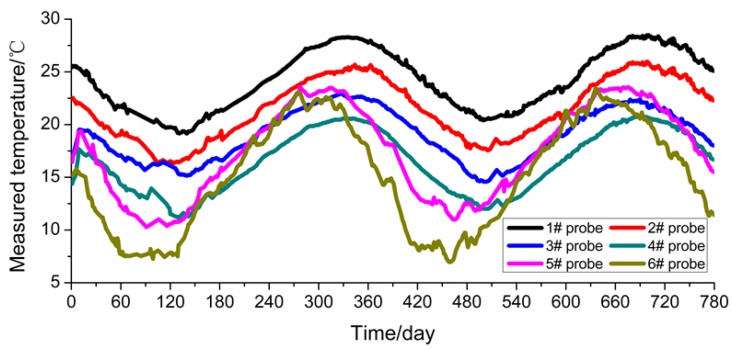
Temperature measured by FDR sensors.

The FDR sensors acquire data once every 2 h and take the average value of the daily monitoring data to make a plot as shown in Figure 5. In this study, the monitoring duration is from 10 November 2013 to 16 December 2015, spanning two test cycles. For plotting convenience, the data when the monitoring started (*i.e.*, 10 November 2013) is taken as the first day. In Figure 8, it can be observed that the temperatures at the measuring points are mainly within the range of 8 °C–28 °C. They show a gradual rising tendency from February to August and a gradual declining tendency from August to the following February, and reach their peak and trough in August and February, respectively, showing that the temperature change is highly consistent with the natural environment conditions. The temperature values measured by the embedded sensors (#5 and #6) close to the outer side of the subgrade slope demonstrate relatively great changes, but those measured by the rest change gently. The reason is that sensors #5 and #6 are closer to the subgrade slope, so they are more influenced by the atmospheric environment.

### 4.2. Moisture Content Observations

**Figure 9:**
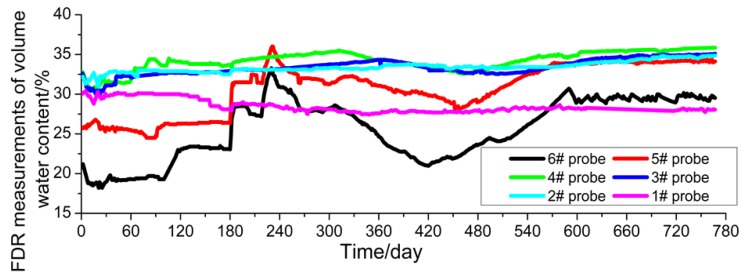
Moisture content measured by FDR sensors.

The change in moisture content (volumetric moisture content) in the subgrade’s soil mass is more complex than the temperature variation measured by the FDR sensors. It can be observed from Figure 9 that the measured values at two points (#5 and #6) on the outside of the subgrade slope show a large change in amplitude, because those two points are subjected to more significant influences from such climatic factors as rainfall, wind speed and evaporation than other points; and they are closer to the outer edge of the subgrade slope and this makes even compaction by the road rollers more difficult, thus leading to uneven compactness of the subgrade. In contrast, the change at the measuring points (#2, #3, #4) in the soil mass of the widened area is relatively gentle, because the soil mass in the widened subgrade is compacted better, with more even compactness (dry density). Even so, the moisture content values at those three measuring points show a slightly rising tendency with time while the results at the measuring points in the old subgrade shows a slightly decreasing tendency with time. This is because when the subgrade is widened, the soil mass of the old subgrade has a higher moisture content than that of the newly-filled subgrade and water migration occurs at the junction of the old and new subgrades to some extent.

### 4.3. Comparative Analysis of Field Measurements and Numerical Simulations

#### 4.3.1. Comparative Analysis of Temperature

**Figure 10:**
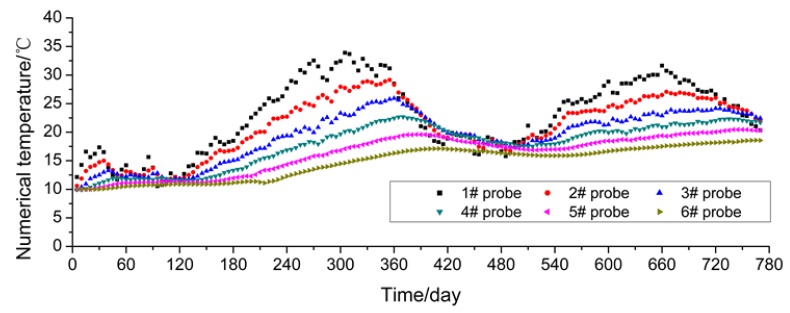
Temperature obtained from the numerical simulation.

The results calculated through the numerical simulation based on the temperature values at the measuring points in the soil mass of the subgrade are plotted in Figure 10. This figure indicates that the soil mass temperature values obtained from numerical calculation show consistency with the natural climate conditions and demonstrate specific periodic changes, and that the values measured by sensors #5 and #6 close to the outer edge of the subgrade slope show a relatively large change amplitude. However, the comparison of measured values (Figure 5) shows that the minimum temperature values from numerical calculation are 16 °C–19 °C around February 2014, which is significantly different from the measured values of 7 °C–20 °C. Further analysis indicates that the reason is that for the numerical calculation it is assumed that the subgrade soil mass is uniform with even compactness and minor porosity. However, in fact, the compactness at different parts of the subgrade and the porosity of the soil mass are not uniform, so pores in the soil mass are easily connected with each other. For the reasons given above, the change amplitude of the temperature values measured by the sensors far from the subgrade slope is smaller than that of the values measured by the sensors close to the outer side of the subgrade slope, which can also be verified by the values measured by the FDR sensors.

**Figure 11:**
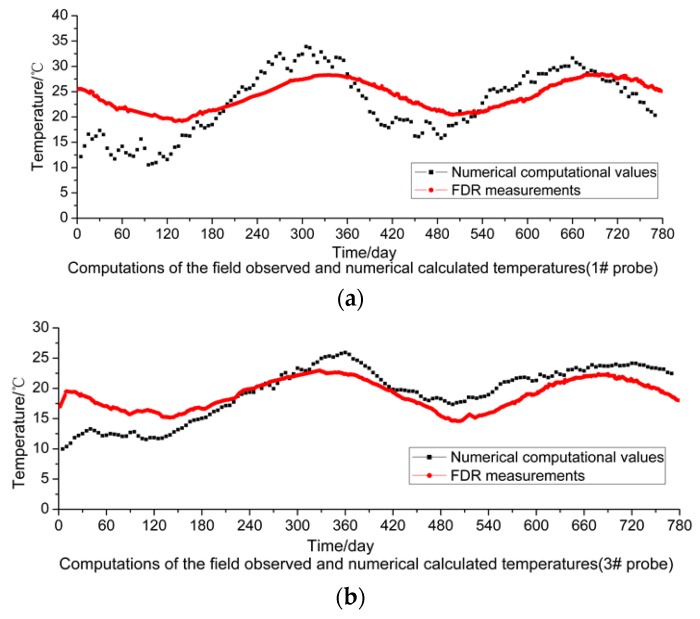

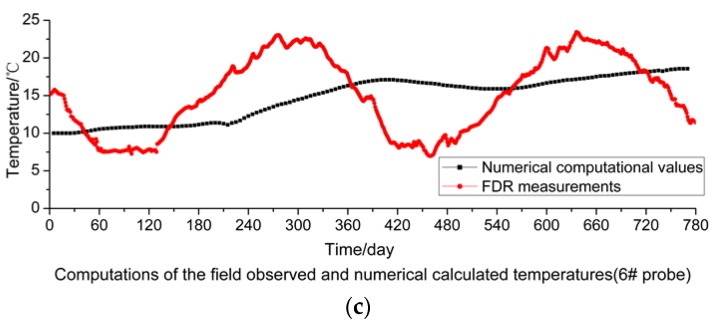
Comparison of measured and calculated temperatures: (**a**) #1 probe; (**b**) #3 probe; (**c**) #6 probe.

Moreover, to better compare and analyze the values measured by the FDR sensors and calculated through numerical simulations, sensors #1, #3 and #6 are taken as the objects for comparative analysis. From Figure 11a–c below, it can be observed that the values measured by sensors #1 and #3 are very consistent with the calculated values both in their change tendencies and change laws. It is because these two sensors are far from the subgrade slope and the measuring points are located in the middle of the widened subgrade, so the road roller can provide uniform compaction. Conversely, for sensor #6 close to the outer side of the subgrade slope, great deviations appear between the measured and calculated values in their change tendencies and amplitudes. The reason for such a difference, as analyzed above, is that the compactness of subgrade soil mass is not uniform and the pores in the soil mass may be interconnected.

To sum up, in terms of the internal temperature of the subgrade soil, the compactness in the middle of the widened subgrade is uniform, so the measured and calculated values are close while the values measured by the FDR sensors close to the outer edge of the subgrade are greatly different from the calculated values. According to the analysis, the values measured by the FDR sensors are more consistent with actual conditions, so it is more reasonable to use measured values for evaluating the internal temperature of subgrade soil.

#### 4.3.2. Comparative Analysis of Moisture Content

**Figure 12:**
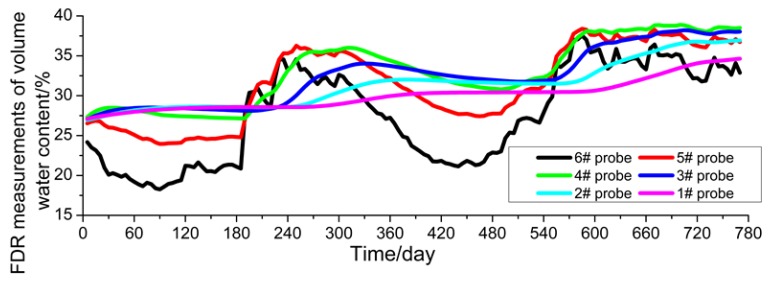
Volumetric moisture content obtained from the numerical simulation.

**Figure 13:**
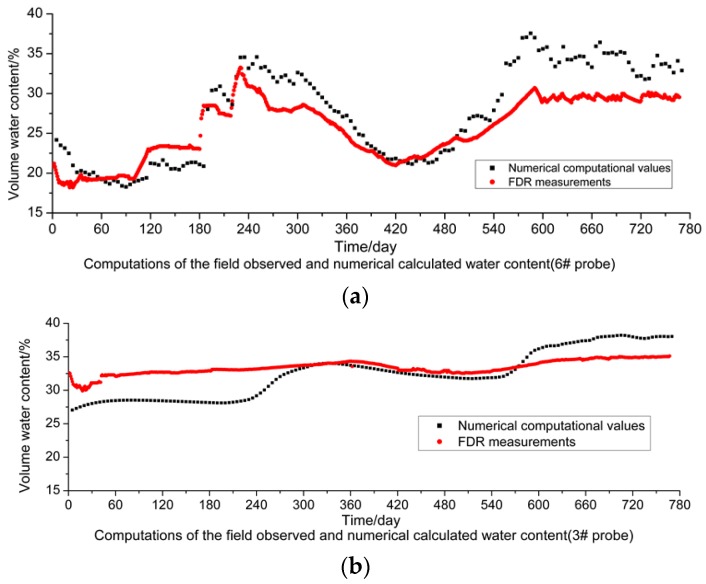

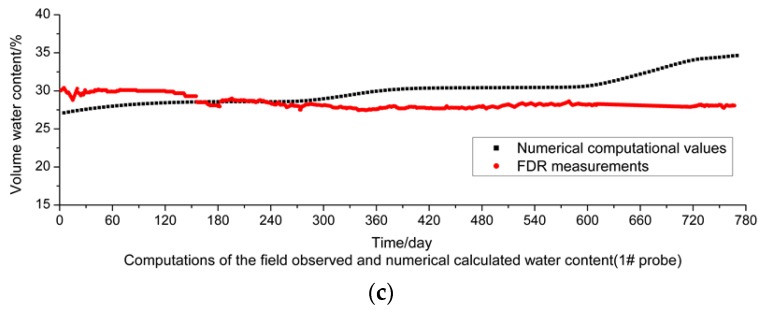
Comparison of measured and calculated volumetric moisture content: (**a**) #6 probe; (**b**) #3 probe; (**c**) #1 probe.

According to the comparison between the values of moisture content in the subgrade soil mass obtained through numerical calculation, plotted in Figure 12, and its measured values (Figure 8), large deviations between them can be found, but favorable consistency is shown in some positions. For the comparative analysis of the values measured by the FDR sensors and calculated through numerical simulations, the sensors #1, #3 and #6 are taken as the objects. From Figure 13a–c, it can be observed that for sensor #6 close to the outer side of the subgrade slope, the measured values are very consistent with the values obtained through numerical calculation and so are their change tendencies. This is because the soil mass close to the outer side of the subgrade is more influenced by the climatic environment, and can more quickly respond to any changes in climatic conditions.

From Figure 13b, the volumetric moisture content values measured by sensor #3 are close to the values obtained through numerical calculations. However, the change amplitude of the moisture content values measured by sensor #3 is obviously lower than that of values measured by the sensor #6. According to careful analysis, this is because probe #3 is 5 m away from the subgrade slope and subject to slighter direct climatic condition influences than sensor #6. Additionally, the change amplitude of the moisture content values measured by sensor #3 is not as large as that of the values obtained through numerical calculation. The reason is given as follows: the increase or decrease of the moisture content in the subgrade soil mass is realized by the infiltration or evaporation of rainwater outside; in numerical calculation, it is assumed that subgrade soil is uniform; however, the subgrade soil mass is actually a heterogeneous body, so the effects of infiltration or transpiration on different parts of the subgrade are not consistent, which gives rise to the inconsistency of moisture content in the subgrade soil mass.

From Figure 13c, for sensor #1 embedded in the old subgrade, the measured values are substantially different from and even inconsistent with the values obtained through numerical calculation. According to deliberate analysis, the reason is that the moisture content in the old subgrade, having been subject to over a decade of operation, increases [13]; however, the newly-widened subgrade is filled under optimum moisture content conditions, so water migrates from the old subgrade to the new one, which causes a further decline of the moisture content in the old subgrade. In contrast, according to the calculated values, the moisture content in the old subgrade rises, because the moisture content values of the old and new subgrades are designed to be same when the mathematical model is established. The discrepancy shown by sensor #1 can more effectively reflect the contrast between the measured values of FDR sensors and the calculated values. In addition, the analysis on actual conditions at the field shows that measured values of FDR sensors are more consistent with the actual conditions, thus further verifying that the values measured by FDR sensors are effective.

## 5. Conclusions

Based on the field monitoring and numerical simulation, the following conclusions can be drawn:

(1) FDR sensors were applied to the subgrade in a highway reconstruction project for the first time, and the embedding method and process of the sensors are illustrated. During a monitoring period of over two years, all embedded sensors present reasonable performance for monitoring the temperature and moisture inside the subgrade soil. This implies that the embedding method and process proposed in this paper are reasonable and also indicates that agricultural-purpose FDR sensors are applicable to the subgrade in highway reconstruction and extension projects.
(2) By means of the small weather station built in the field and the GPRS module performing wireless transmission, the real-time data monitored by FDR sensors can be acquired remotely. Thus, the labor cost can be largely reduced and analysis of real-time data monitored is accessible, thereby enhancing timely and effective evaluation of subgrade performance.
(3) Comparison between the measured results by FDR sensors and computed results from numerical simulation also demonstrated that the FDR sensor can effectively reflect the temperature and moisture changes inside subgrades, especially at the junction between the old and new subgrades, the measured values are more reasonable and effective than the calculated values, and can reflect the temperature and moisture changes caused by the climatic environment more accurately.
(4) The paper provides a new method and idea for highway researchers to monitor long-term changes of the temperature and moisture inside the subgrade, and further inspect its long-time performance. This method can be also extended to railway embankments, prevention and treatment of slopes foundation engineering and other civil engineering projects which are affected by soil moisture content and temperature.

## Figures and Tables

**Table 1:** Temperature and moisture content results of calibration.

Name	Function Relationship	Range of Application
Relationship between Soil volumetric water content and voltage	θ = 144.7 υ^4^ − 285.0 υ^3^ + 195.0 υ^2^ − 5.277 υ	Υ ≤ 1.005 V
	θ = 500.0 υ − 452.5	1.005 V < υ ≤ 1.085 V
	θ (% vol) = 100	1.085 V < υ
Relationship between soil temperature and voltage	T = 10.312 × (υ/100) − 81.25	−30 °C~+50 °C

θ—water volume content (% vol); υ—sensor output voltage (V); T—temperature (°C).

**Table 2:** Parameters required for the numerical simulation.

Parameter Category	Relevant Parameter	Symbol	Unit
Meteorological parameters	Daily average temperature	T	°C
	Daily relative moisture	*RH*	%
	Daily relative wind speed	*u*	m/s
	Daily average rainfall	*P_r_*	mm
Hydraulic properties of soil	Saturated infiltration coefficient	*k_ws_*	m/s
	Soil-Water Characteristic Curve	SDSWCC	-
Thermodynamic properties of soil	Heat conductivity coefficient	*λ_t_*	-
	Specific heat per unit volume	λv	J/(m^3^·°C)
Physiological parameters of vegetation	Leaf area index	*LAI*	-
	Root depth index	*D_R_*	m

**Table 3:** Fitting results of soil water characteristic curve.

Material	*a*	*n*	*m*	*S_r_*	*θ_s_*	R^2^
**Low Liquid-Limit Clay**	**129.331**	**1.476**	**0.555**	**10^6^**	**40.32%**	**0.973**

**Table 4:** Hydraulic and thermal property parameters of subgrade soil.

Materials	Initial Degree of Saturation (%)	Infiltration Coefficients (m/s)	Soil Water Characteristic Curve Parameters			Thermal Conductivity Coefficient	Volumetric Heat Capacity (J·m^−3^)
			a	n	m		
**Foundation**	Value of groundwater	4.75 × 10^−6^	72.01	1.62	0.48	2.742	**2.76 × 10^6^**
**low liquid-limit clay**	75	1.15 × 10^−8^	129.33	1.48	0.56	2.354	**2.85 × 10^6^**
**Asphalt pavement**	-	**1.0 × 10^−14^**	-			**1.010**	**1.98 × 10^6^**

## Appendix A

**Table A1:** Main technical parameters of FDR probe.

Project	Indicators	Note
Measurement parameters	Measured medium volumes of moisture content	-
Application objects	Soils, other solids or powdered medium	-
Accuracy of measurement	±0.5% vol ((if the soil was calibrated) ±2.0% vol (direct measurement)	Range of 0~saturated water content
Resolution ratio	±0.1% vol	-
Deviation caused by Salinity	Not more than 3.5% vol (0 saturated moisture content)	FDR can be used after calibrating
Power consumption	22 mA (characteristic value)	-
Supply voltage	9–12 V	-
Output signal	0–1200 mV	-
Measurement of volume	60 mm (length) × 30 mm (diameter)	-
Work environment	−30 °C~+50 °C	-
